# Supplementary material for: Activation of Endothelial Large Conductance Potassium Channels Protects against TNF-α-Induced Inflammation
Source: Int J Mol Sci. 2023 Feb 17;24(4):4087. doi: 10.3390/ijms24044087 (PMC9961193; doi:10.3390/ijms24044087)
Supplement: Supplementary file 1 [file ijms-24-04087-s001.zip › ijms-2020358-supplementary.pdf]

Supplemental Figure S1

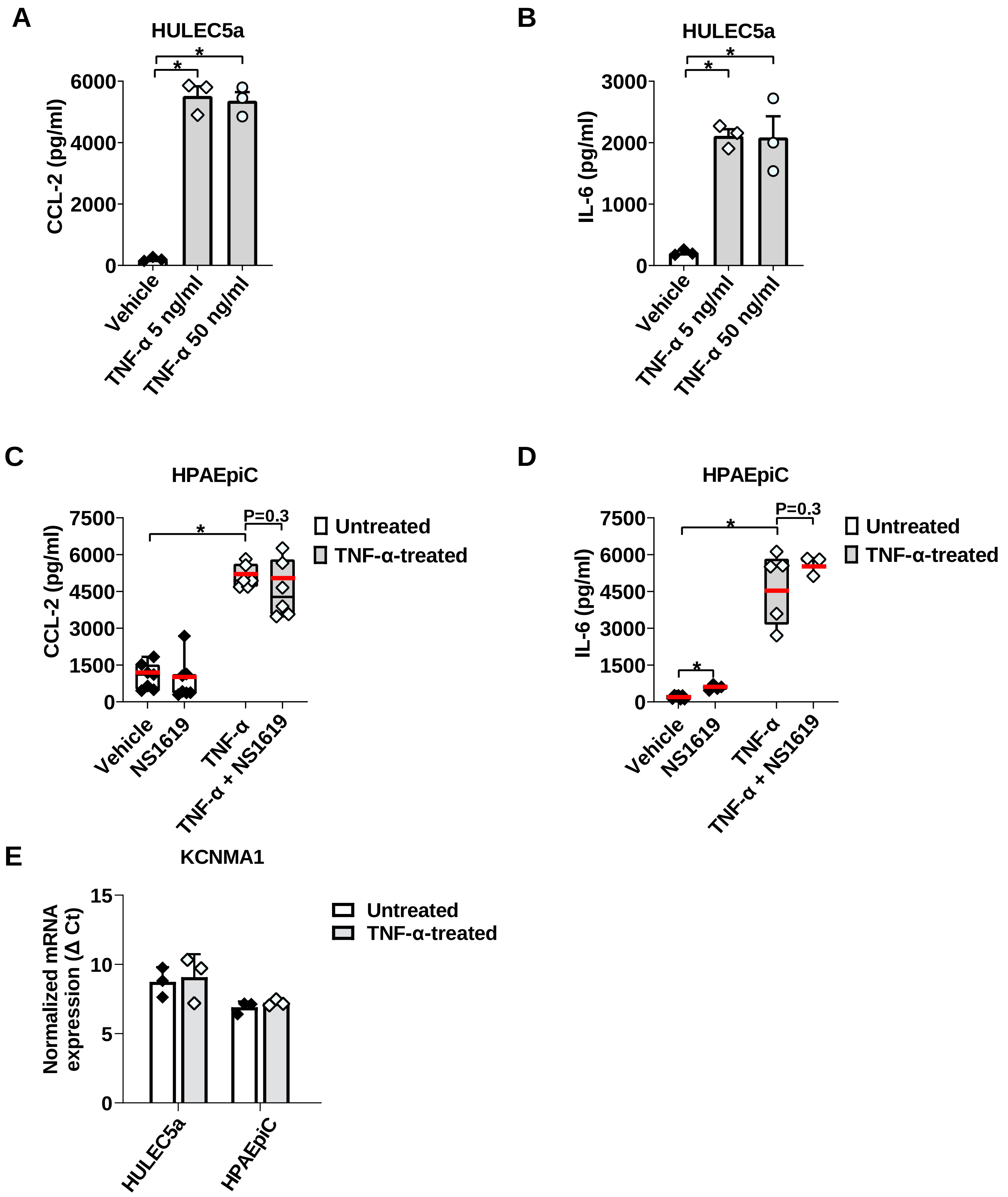

Supplementary Figure S1. (**A**,**B**) TNF-α dose-response experiments. Pilot ELISA experiments using HULEC5a cells reveal that 5 ng/mL and 50 ng/mL of TNF-α evoke a similar increase in CCL-2 and IL-6 levels. (**C**,**D**) TNF-α treatment (5 ng/mL; 24 h) also increases CCL-2 and IL-6 secretion form primary human alveolar epithelial cells (HPAEpiC), while BK activation with NS1619 has no effect on CCL-2 or IL-6 secretion from these cells (n = 3–11, * $p \leq 0.05$). Mean values are represented in red horizontal bars; black bars indicate medians. (**E**) RT-PCR results show that TNF-α treatment does not affect BK channel gene expression in either pulmonary microvascular endothelial (HULEC5a) or primary alveolar epithelial (HPAEpiC) cells (n = 3). Bars represent mean values + SEM. n = number of separate cell passages, which are considered as biological replicates.
